# Development Process and Evaluation of an Anti-racism Primer and Toolkit for Medical Educators

**DOI:** 10.7759/cureus.88188

**Published:** 2025-07-17

**Authors:** Meghan T O'Brien, Rachel Fields, Andrea Jackson, Megha Garg

**Affiliations:** 1 Internal Medicine, University of California San Francisco, San Francisco, USA; 2 Family Medicine, Sutter Medical Group, Sacramento, USA; 3 Obstetrics, Gynecology, and Reproductive Sciences, University of California San Francisco, San Francisco, USA

**Keywords:** anti-racism, curriculum development, diversity, equity, inclusion, medical education

## Abstract

Introduction: Medical educators are expected to address racism and health inequities in their curricula but may feel ill-equipped to do so. Currently, resources to support educators in reviewing and revising curricula through an anti-racism lens are limited. We describe the development process and evaluation of an anti-racism primer and toolkit for medical educators to support curriculum development.

Methods: In 2020, three members of the University of California, San Francisco Differences Matter Educational Action Group collaborated to create an iterative, self-directed tool to support educators in creating and revising teaching materials to be anti-racist: the Anti-Racism and Race Literacy: Primer and Toolkit for Medical Educators (Anti-Racism Primer). From August 2020, all medical educators in pre-clinical coursework were recommended to use the Anti-Racism Primer for their curricular materials and asked to complete a feedback survey with questions related to the primer's effectiveness for anti-racism knowledge and curricular revisions.

Results: We received 82 survey responses from 40 content creators and 42 facilitators. Twenty-seven (67.6%) of content creators use the toolkit to revise curricular materials. Twenty (74.1%) content creators who used the toolkit strongly agreed that the Anti-Racism Primer helped them reflect on their materials. Over half (55.6%) strongly agreed they made revisions using the Anti-Racism Primer, and a third felt it made their materials more equitable. Respondents described examples of revisions they made, including increased inclusive language, eliminating racial stereotypes, diversification of photos, and more precise use of genetic ancestry to describe population risk.

Conclusion: The Anti-Racism Primer is an effective resource for medical educators to support their understanding and incorporation of anti-racism into curricular materials. Dissemination as an institutionally supported resource supports utilization and can amplify pre-existing efforts toward equitable change. Future steps include studies about the impact of such resources on learners and further evaluation of faculty development needs.

## Introduction

Medical educators and clinicians are often called upon to address race, racism, and health disparities while teaching and delivering care [1,2]. There is increased recognition that medical school curricula can perpetuate false beliefs and implicit biases and lead to misdiagnosis through stereotyping, lack of representation, and racism. This, in turn, can negatively impact clinical care and drive health disparities [3-12]. These factors also affect the integrity and safety of the learning climate and may impact learner success [13]. There is increasing recognition that the physician workforce needs to understand the complex mechanisms, contexts, and manifestations of racism to decrease health disparities and prevent harm to patients and learners [2,14]. However, many educators feel unprepared to address race and racism in curricular materials or the learning environment [15, 16]. As a result, learners may receive an education that inadvertently reinforces biases and inequitable structures in health care.

Recognizing these educational failings, and sparked by the decision not to indict White police officers involved in the murders of unarmed Black men in 2014, medical students around the country organized protests to call attention to how racism is reinforced institutionally and in education. In response to the medical student organization White Coats for Black Lives led a die-in to demand institutional action, the University of California, San Francisco School of Medicine (UCSF) established the Differences Matter Initiative to advance diversity, equity, and inclusion, with a $10 million commitment over five years [17,18]. Through this initiative, a multidisciplinary Education Action Group identified a need to support faculty in revising and creating anti-racist educational materials. 

Although neither one’s race nor individual experience of racism necessarily confers comfort or expertise in addressing racism, those with identities historically excluded [19] from medicine are frequently called upon to advance diversity, equity, inclusion, and belonging. This work is under-recognized and often uncompensated, reflecting a disproportionate burden, or “minority tax” [20]. Requests to provide feedback on or revise curricular materials are one manifestation of this tax. To reduce the minority tax and improve the quality of medical education, resources are needed to guide all educators to examine and revise their curricular materials to disrupt inadvertent racism [21].

Working as part of the Differences Matter Educational Action Group, we developed a comprehensive guide entitled the “Anti-Racism and Race Literacy: Primer and Toolkit” (Anti-Racism Primer) to address the gap in peer-reviewed resources for faculty and meet the needs of educators at our institution. Currently, few resources in the peer-reviewed literature exist to support faculty as they develop curricula conscious of bias, stereotypes, structural racism, and health disparities [22]. We identified one checklist for self-assessment to identify and ameliorate areas of bias [23]; however, it assumed the user already had the foundational knowledge needed to understand and implement the necessary revisions.

Because checklists have become an industry standard to achieve higher baseline performance through a standardized approach [24], we included a checklist for educators to guide their self-review, as well as accompanying examples of checklist applications. The checklist helps to identify and revise biased representations, disrupt normative perceptual frames, center health disparities, and interrogate the methodology and objectivity of data to cultivate critical reasoning.

Objectives

We describe the development process and evaluation of an anti-racism primer and toolkit for medical educators to support curriculum development. Specific areas of feedback from educators included whether the Anti-Racism Primer facilitated reflection on and revision and design of curricular materials. 

## Materials and methods

Anti-racism primer development process 

Our toolkit was created for medical school faculty at UCSF, with a student body of approximately 600 medical students. In 2018, the authors (MTO, RF, AJ), working as part of UCSF’s Differences Matter Educational Action Group, developed the Anti-Racism Primer for Medical Educators [25]. The authors were two women faculty medical educators, one Black and one White, and one White woman medical student.

In alignment with Kern’s six-step approach [26] to curriculum development, we identified faculty needs through peer discussions, aggregated student feedback on curricular content, and unpublished internal qualitative research on faculty experience with race and racism in their curriculum and classrooms. Then, we established goals and objectives and developed an educational resource for self-directed application to curricular materials. During the development process, we incorporated iterative feedback from Educational Action Group members, colleagues, and students with expertise in equity and education, and educators who might use the resource. To balance the goals of responsibly handling complex content and producing a tool that was accessible in length, we created multiple points of entry with clear sections that users could access individually as needed.

The Anti-Racism Primer includes an introduction that reviews the reasons for educators to address racism in their materials, the objectives of the primer and toolkit, and instructions for use. It was emphasized that the Anti-Racism Primer provided a starting point for continued self-development.

The Anti-Racism Primer was divided into four steps: 1) Prepare to talk about rRacism and race, which helped navigate the tensions and emotions that surface when discussing these topics; 2) Definitions and frameworks, which provided a mental model of race as a social construct distinct from genetic ancestry; 3) Historical origins of racism in healthcare and medicine, which delved into how the medical system reinforced racism and incorporated racist ideas into its structure; and 4) Implement anti-racism in medical rducation, which provided a checklist guide for developing anti-racist materials and revising materials. Each section was designed to stand alone and included reflection questions and a list of additional resources for deeper engagement.

In July 2020, the Anti-Racism Primer was launched as a living document, updated once per year or as needed by an author (MO) to reflect emerging literature, on the UCSF Differences Matter website [25] as a resource for UCSF health professions educators. Those outside of UCSF could also access and download the document from the publicly accessible website.

At the time of launch, UCSF’s Office of Medical Education asked interdisciplinary course directors and educators involved with the preclinical and clerkship curricula for first- and second-year medical students to review the Anti-Racism Primer and use it to revise their curricular materials.

Anti-racism primer evaluation 

After curricular blocks between November 2020 and July 2021, educators who were offered the resource received a survey via email, with a reminder sent two weeks after the initial email, to assess their experience using the Anti-Racism Primer. Following Kern's six-step methodology [26], we evaluated this educational intervention with a survey consisting of multiple-choice questions, Likert scale questions, and open-ended questions about the individual components of the Anti-Racism Primer (Appendix A). We defined “effectiveness” of the Anti-Racism Primer broadly as the majority of users finding benefit from the toolkit in curriculum development across the array of questions asked in our survey. The open-ended questions were intended to give context to multiple-choice question answers and understand specific curricular changes, and were not designed for thematic qualitative analysis.

## Results

We received 82 complete survey responses from 1,314 UCSF faculty course directors and health professions educators (response rate 6.2%). Seventy-eight (95.1%) respondents were from the Department of Medicine, with eight (9.8%) from the School of Pharmacy, three (3.7%) from the School of Nursing, four (4.9%) from the School of Dentistry, and one (1.2%) other (participants could select multiple affiliations). Fifty (61.0%) of respondents identified as White, 15 (18.3%) as Asian, five (6.1%) as African American, and three (3.7%) as Native Hawaiian or other Pacific Islander, while nine (11.0%) preferred not to say or to self-identify (participants could select all races that applied). Fifty-one (62.2%) respondents identified as female and 29 (35.4%) as male. Eleven (13.4%) identified as lesbian, gay, bisexual, transgender, queer or questioning, intersex, or asexual or ally (LGBTQIA), while 66 (80.5%) did not, and five (6.1%) preferred not to say. Thirty-four (41.5%) had been involved in health professions education for <5 years. Respondent characteristics are reflected in Table 1.

**Table 1:** Respondent characteristics LGBTQIA: lesbian, gay, bisexual, transgender, queer or questioning, intersex, and asexual or ally

Participant characteristics	N= 82 (%)
Gender	
Female	51 (62.2%)
Male	29 (35.4%)
Non-Binary	0 (0.0%)
Prefer to self-identify	0 (0.0%)
Preferred not to say	2 (2.4%)
Race	
American Indian or Alaska Native	0 (0.0%)
Asian	15 (18.3%)
Black or African American	5 (6.1%)
Hispanic, Latino, or Spanish origin	8 (9.8%)
Native Hawaiian or other Pacific Islander	3 (3.7%)
White	50 (61.0%)
Prefer to self-identify	4 (4.9%)
Prefer not to say	5(6.1%)
LGBTQIA	
Yes	11 (13.4%)
No	66 (80.5%)
Prefer not to say	5 (6.1%)
Raised in the United States	
Yes	66 (80.5%)
No	13 (15.9%)
Prefer not to say	3 (3.7%)
Years in medical education	
<5	34 (41.5%)
5-10	14 (17.1%)
10-15	19 (23.2%)
>15	15 (18.3%)

Overall, 48 (58.5%) of respondents reviewed the Anti-Racism Primer, while 20 (24.4%) did not use it, and 14 (17.1%) reported never receiving it. Forty (48.8%) of the 82 respondents contributed course content, while 42 (51.2%) did not (i.e., facilitators). Of the 40 content contributors, 27 (67.5%) reviewed the toolkit, compared to 21 (50%) of the 42 educators who did not contribute content.

**Figure 1:**
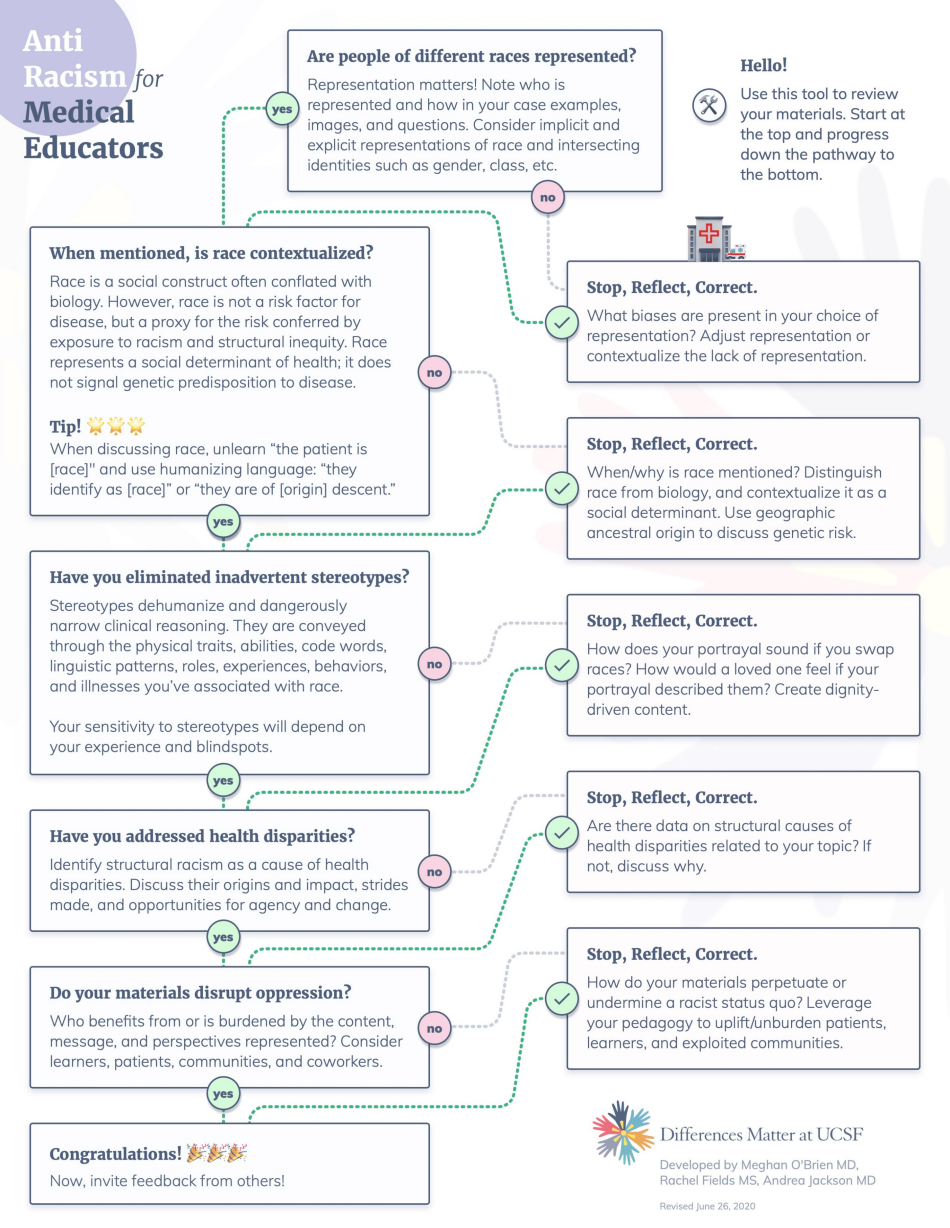
One page anti-racism checklist for medical educators

Content creators used the toolkit to revise various curricular materials, including lectures/PowerPoints (22/27, 81.5%), small-group session guides (19/27, 70.4%), clinical vignettes or cases (15/27, 55.6%), exam/practice questions (7/27, 25.9%), and something else (5/27, 18.5%) (respondents could select all that applied). The most used sections of the Anti-Racism Primer were the “one-page flow chart” (22/27, 81.5%) (Figure 1), “Step 2: Definitions and frameworks” (19/27, 70.4%), “Step 3: Understand race in the historical context of healthcare and medicine” (17/27, 63.0%), and “Step 1: Prepare to talk about racism and race” (14/27, 52.9%). Other sections were used less frequently, including “Applying the guide: examples” (11/27, 40.7%), “Introduction” (9/27, 33.3%), and “Additional resources for exploration” (4/27, 14.8%).

**Figure 2:**
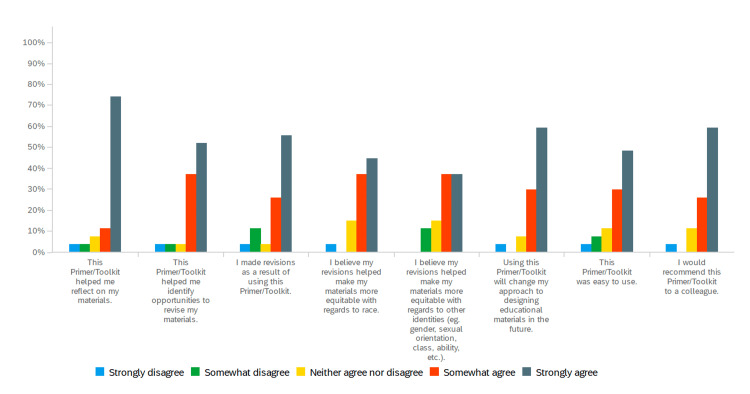
Experience using the Anti-Racism and Race Literacy Primer and Toolkit for Medical Educators

Considering their experience using the Anti-Racism Primer, 20/27 (74.1%) of content creators strongly agreed it helped them reflect on their materials, and 16/27 (59.3%) strongly agreed it would change their approach to designing educational materials in the future and identify opportunities to revise materials. A majority, 15/27 (55.6%), strongly agreed that they made revisions because of using the Anti-Racism Primer. Ten of 27 (37.0%) somewhat agreed and 10/27 (37.0%) strongly agreed that the revisions made their materials more equitable. Thirteen of 27 (48.2%) strongly agreed and 8/27 (29.6%) somewhat agreed that the Anti-Racism Primer was easy to use. Sixteen of 27 (59.3%) strongly agreed and 7/27 (26.9%) somewhat agreed that they would recommend it to a colleague (Figure 2).

Our evaluation survey collected reflective statements from participants regarding the types of changes they made as a result of using the Primer. Respondents who created content described examples of revisions they made because of using the Anti-Racism Primer (Table 2). 

**Table 2:** Examples of changes made because of using the Anti-Racism Primer DEI: diversity, equity, and inclusion

Theme	Example
Language	Used more inclusive language. Removed racial stereotypes. Reconsidered and revised sex- and race-based language. Removed stereotypes and outdated language (e,g, “third world”)
Representation	Reviewed and modified vignettes. Included more photos of skin of color. Included multiple gender identities in examples
Content	Added different readings focused on equity. Incorporated a focus on anti-racist medicine. Added slides about disparities in care (race, gender, sexual orientation
Centering equity	Incorporated statement about educator’s DEI and anti-oppressive goals. Incorporated statement affirming values, incorporation of health equity and access. Created a new curriculum centering on health equity. The course directory asked lecturers to use a primer/toolkit when creating content
Uncoupling race/genetics	Looked deeper into the evidence provided to support differences/disparities based on race. Removed language that suggested race has a biological basis. Distinguished risk factors based on genetic ancestry and structural factors from race. More careful references to genetic ancestry and populations. When incorporating data that are published using race as a proxy for genetics, reflected on whether the data is pertinent. Challenges: Distinguishing race from genetics does not reflect how genetics is implemented in practice Difficult to incorporate data from studies that use race as a proxy for genetics
Reinforcement	Use the primer as a source to advocate for changes in content that were previously resisted

These included increased use of inclusive language, eliminating racial stereotypes, diversification of photos, including more expansive gender representation, inclusion of values-affirming statements in syllabi, incorporating health equity topics and evidence, removal of language suggesting race is biological, distinguishing risk factors of genetic ancestry from structural risk factors, and more precise use of genetic ancestry to describe population risk. Others described greater awareness of language used, having an easier time discussing racism with learners, and opening more space in sessions for learner perspectives. Another respondent described that having an institution-endorsed resource empowered their prior advocacy for curricular changes that previously received pushback. Critiques included that the Anti-Racism Primer was too long and bulky. Others noted the difficulty of incorporating discussion points about how race and genetics are distinct when the studies they teach historically conflated the two by using race as a proxy for genetic ancestry.

Those respondents who did not contribute content but reviewed the Anti-Racism Primer to facilitate sessions with students described it as helpful for more specific anti-racism vocabulary, awareness of the magnitude and extent of systemic racism in our society, and language and awareness to bring more nuance to conversations.

## Discussion

We developed the Anti-Racism Primer to disseminate a shared mental model and approach to handling the topics of race and racism in educational content. The goal was to offer students a more consistent educational experience that minimized harm and prepared them to respond to health disparities. Anti-Racism Primer users agreed that the resource helped them make revisions that improved perceived equity in their curricular materials. Many described concrete changes they made after engaging the toolkit or feeling better equipped to discuss race with more precision and nuance. At least one respondent noted that its dissemination as an institutionally supported resource reinforced their prior unheeded advocacy for curricular change, suggesting some easing of the minority tax [20]. The importance of institutional support in resource adoption was paramount. 

We navigated a tension between responsibly handling complex content and producing a tool accessible in length. To address this, we made multiple entry points with clear sections that users could access individually to suit their needs. Facilitators more commonly used the background information sections, while content creators more commonly used the toolkit checklist components, reflecting the success of this approach. We chose the format of a living document, allowing for responsiveness to both educators’ dynamic needs, new concerns, and evolving understandings of the mechanisms and manifestations of racism. Alternative formats that are shorter, delivered as faculty training workshops, or interactive formats could make the toolkit more accessible. An institutional expectation that the Anti-Racism Primer would be reviewed by educators interacting with students supported its use.

While the toolkit and primer focus on race, we recognize that the depiction and treatment of other identities, including, but not limited to, gender, age, sexuality, ability, education, and economic status, also require thoughtfulness and skill. We choose to center on understanding race and racism because racial inequities are deeply rooted, pervasive, and traverse all indicators of success when other aspects of identity are controlled [27]. 

The Anti-Racism Primer and our evaluation of its efficacy have limitations. We are a large, publicly funded institution with multiple initiatives devoted to advancing diversity, equity, inclusion, and belonging. This created a climate of receptivity among educators, the lack of which may limit adoption in other settings. Survey respondents continued to struggle with the tension between genetics and race as a proxy for genetics, which we did not discuss as deeply in the Anti-Racism Primer due to length considerations. Our survey assessed educator perceptions of the Anti-Racism Primer and perceived improvement in curricula, which reflect neither the learners’ experience of those materials nor objective curricular material review.

Limitations of the survey include selection bias of respondents (people who used the toolkit were more likely to respond) and a low response rate. The low response rate has several underlying considerations. First, the survey was sent to all educators, whether they facilitated one session or created course content. This range of faculty may have led to many not remembering the Anti-Racism Primer or choosing to respond to the survey request. There was also a lag of months from when some faculty used the primer to when they received the related survey. However, we felt it was important to send the survey out widely and not bias the responses by targeting specific educators. Finally, keeping the Anti-Racism Primer up to date with evolving knowledge and terminology is challenging. Further resources are required to support ongoing revisions. 

The Anti-Racism Primer addressed a knowledge gap within our institution where few external resources existed at the time. The checklist from the Anti-Racism Primer has been adapted to a faculty development workshop focused on equitable approaches to curriculum development. It has also been adapted, in conjunction with materials from UCSF’s Anti-Oppressive Curriculum Initiative, into a content review checklist that Office of Medical Education staff use to support workshop creators in revising their content to be anti-oppressive. This Anti-Racism Primer contributes to a growing number of resources for developing anti-racist curricula [28-30]. Future directions for these resources include focused support tools for specific, more challenging, and controversial content areas (i.e., genetics) in partnership with content experts. In particular, regarding such toolkits, more robust studies including comparison or control groups and direct review of curricular materials rather than educator perceptions would help with understanding the direct impact of such resources on curricula. 

## Conclusions

The Anti-Racism Primer is an effective resource to support health professions educators in their understanding and incorporating anti-racism in the creation and revision of curricular materials. Users engaged with different sections depending on their needs, and most described that using the Anti-Racism Primer supported reflection and would influence their approach to future content development. Dissemination as an institutionally supported resource supports utilization and can amplify pre-existing efforts toward equitable change. Future steps include studies about the impact of such resources on learners and further qualitative evaluation of faculty development needs in anti-racism education.

## Appendices

Appendix A 

Qualtrics Survey for Anti-Racism Primer and Toolkit Users

**Table 3:** Qualtrics Survey for Anti-Racism Primer and Toolkit users.

Welcome! We are conducting a quality improvement survey to understand more about your experience using the Anti-Racism and Race Literacy Primer and Toolkit for Medical Educators and the impact its use had on your curricular materials In this survey, we will ask you about your experience using the Primer and Toolkit and to optionally submit your original and revised educational materials (ideally with changes tracked) so that we can learn more about the types of changes the tool prompts educators to make. This survey is best done from a computer rather than a phone. If you choose not to upload your materials, you can still complete the rest of the survey. Your survey responses and any submitted materials will be kept confidential within the study team. The survey will take approximately 5-10 minutes. Your participation in this study is voluntary and your consent to participate may be withdrawn at any time. For questions or concerns, please contact [email]. By clicking the button below, you acknowledge: Your participation is voluntary. You are 18 years of age. You are aware that you may choose to terminate your participation at any time for any reason.	
Question	Answer choices
Did you develop any content for the course? E.g., lecture, small group session guide, clinical vignettes, etc.	-Yes -No
Did you review the Anti-Racism and Race Literacy Primer and Toolkit for Medica lEducators for this course?	-Yes -No - I have not received the Toolkit
Content Creators	
How many times have you reviewed and revised educational materials using the Anti-Racism and Race Literacy Primer and Toolkit for Medical Educators?	-This is my first time using the materials -Once before -Twice before -Three or more times
What type of educational materials did you review and revise using the Anti-Racism and Race Literacy Primer and Toolkit for Medical Educators? Select all that apply.	-Lecture, powerpoint -Exam, practice questions -Clinical vignettes or cases -Small group session guide -Study guide -Something else (free text)
Which sections of the Anti-Racism and Race Literacy Primer and Toolkit for MedicalEducators did you use? Select all that apply.	-Introduction -Step 1: Prepare to talk about racism and race -Step 2: Definitions and Frameworks -Step 3: Understand race in the historical context of health care and medicine -Step 4:Implement anti-racism in medical education (including longer Guide for --Developing Anti-Racist Teaching Materials) -One page flow chart -Applying the Guide: Examples -Additional Resources for Exploration
Consider your experience using the Anti-Racism and Race Literacy Primer and Toolkit for Medical Educators. Please indicate the extent to which you agree with the following statements: -The Primer/Toolkit helped me reflect on my materials -The Primer/Toolkit helped me identify opportunities to revise my materials -I made revisions as a result of using the Primer/Toolkit -I believe my revisions helped make my materials more equitable with regards to race -I believe my revisions helped make my materials more equitable with regards to other identities (eg. gender, sexual orientation, class, ability, etc) -Using the Primer/Toolkit will change my approach to designing educational materials in the future. -The Primer/Toolkit was easy to use -I would recommend the Primer/Toolkit to a colleague.	Likert scale: Strongly disagree, somewhat disagree, neutral, somewhat agree, strongly agree
Please describe the changes you made to your curricular materials as a result of reviewing and revising them using the Anti-Racism and Race Literacy Primer and Toolkit for Medical Educators.	Free text response.
What additional feedback or comments do you have on the Anti-Racism and Race Literacy Primer and Toolkit for Medical Educators? Consider your experience using the materials and the materials themselves.	Free text response.
Please upload your ORIGINAL materials here. We are very interested in seeing your teaching materials; however, if you cannot upload your materials, you can still complete the rest of the survey.	Upload option.
Please upload your REVISED materials here (ideally with changes tracked) reflecting the revisions you made using the Anti-Racism and Race Literacy Primer and Toolkit for Medical Educators. We are very interested in seeing your teaching materials, however, if you cannot upload your materials, you can still complete the rest of the survey.	
Facilitators	
Which sections of the Anti-Racism and Race Literacy Primer and Toolkit for Medical Educators did you use? Select all that apply.	-Introduction -Step 1: Prepare to talk about racism and race -Step 2: Definitions and Frameworks -Step 3: Understand race in the historical context of health care and medicine -Step 4:Implement anti-racism in medical education (including longer Guide for --Developing Anti-Racist Teaching Materials) -One page flow chart -Applying the Guide: Examples -Additional Resources for Exploration
What impact did the Primer and Toolkit have on your teaching experience?	Free text response.
Educational Affiliation	
How long have you worked in medical or health professions education?	<5 years 5-10 years 10-15 years >15 years
What is your current academic affiliation?	UCSF Other (free text)
Which of the following describe your professional role(s)? Select all that apply.	Administrator Basic Scientist Clinician Educational Scholar Educator Hospital Leadership Social Scientist Trainee (intern, resident, fellow) Training program leadership Other (free text)
With which health professions school are you affiliated? Select all that apply.	School of Dentistry School of Medicine School of Nursing School of Pharmacy School of Physical Therapy School of Social Work Other (free text)
What level are your learners? Select all that apply.	Undergraduate Medical Education Graduate Medical Educaiton Continuing Medical Education Other (free text)
Demographics	
Because our sensitivity to racism and inequity may be impacted by our lived experience and the identities we hold, we would like to understand more about your identities. Please answer the following optional questions. How do you identify in terms of race? Optional. Select all that apply. (For research purposes we are using the AAMC demographic categories, which we acknowledge are limited.)	-American Indian or Alaska Native -Asian -Black or African American -Hispanic, Latino, or Spanish Origin -Native Hawaiian or Other Pacific Islander -White -Prefer to self-identify: (free text) -Prefer not to say
What is your gender? Optional. Choose all that apply.	-Genderqueer or non-binary -Man -Woman -Prefer to self identify: (free text) -Prefer not to say
Do you identify as a member of the LGBTQIA community? Optional.	Yes No Prefer not to say
Did you grow up in the United States? Optional.	Yes No Prefer not to say
